# Chromatin diminution in Copepoda (Crustacea): pattern, biological role and evolutionary aspects

**DOI:** 10.3897/CompCytogen.v8i1.5913

**Published:** 2014-01-09

**Authors:** Andrey Grishanin

**Affiliations:** 1 Papanin Institute for Biology of Inland Waters Russian Academy of Sciences 152742, Borok Yaroslavl Prov., Russia; 2 Dubna International University for Nature, Society and Man, 141980, Dubna, Moscow Prov., Russia

**Keywords:** Chromatin diminution, evolution, Cyclopoida, Copepoda, Crustacea

## Abstract

This article provides an overview of research on chromatin diminution (CD) in copepods. The phenomenology, mechanisms and biological role of CD are discussed. A model of CD as an alternative means of regulating cell differentiation is presented. While the vast majority of eukaryotes inactivate genes that are no longer needed in development by heterochromatinization, copepods probably use CD for the same purpose. It is assumed that the copepods have exploited CD as a tool for adaptation to changing environmental conditions and as a mechanism for regulating the rate of evolutionary processes.

## The pattern of chromatin diminution in Cyclopoida (Copepoda, Crustacea)

Chromatin diminution (CD) in Cyclopoidais the removal of part ofthe chromosomal material from cells of the somatic cells line in one or two sequential cleavage divisions, while germ-line cells retain their nuclear DNA unchanged throughout ontogeny (Beermann 1977, Grishanin et al. 1996, 2006b, Akifyev and Grishanin 2005). CD in freshwater copepods was initially treated as a pathological event (Amma 1911) or as a manifestation of extranuclear DNA synthesis (Stich 1954, 1962). Later study of the marine copepods *Pseudocalanus* Boeck, 1872 revealed a large number of Feulgen-positive material (Robins and McLaren 1982). This material is concentrated by the division spindle prior to the first cleavage division and becomes dispersed soon afterwards. During the second cleavage division about one tenth of Feulgen-positive material is reduced. Robins and McLaren (1982) noted that the phenomenon of loss of nuclear DNA for marine copepods is not like the CD in freshwater copepods, because, despite the elimination of chromatin, during the first two maturation divisions of the embryo, reduction of the genome is not observed in the somatic cells line. They also suggested that the main cause of fluctuations in the nuclear DNA content in marine copepods is to maintain the ratio of nuclear DNA to the size of the nucleus, which is probably related to body size and the speed of development. Later study found a correlation between the size of the somatic genome and the rate of development for the marine copepods (McLaren et al. 1989).

The phenomenon of chromatin diminution (CD) has been discovered in 23 species of freshwater copepods (Table 1). The timing of CD is species-specific and occurs during one or two cell cycles in early embryogenesis. Numerous studies of CD have shown that during early embryonic cells divisions, somatic cells lose from 45% to 94% of DNA whereas germ line cells preserve the initial amount of DNA *Cyclops furcifer* Claus, 1857, *Cyclops strenuus divulsus* Lindberg, 1957, *Cyclops strenuus strenuus* Fisher, 1851 and *Mesocyclops edax* Forbes, 1891 during prophase of their first meiotic division (Beermann 1966, 1977, Chinnappa 1980, Wyngaard and Chinnappa 1982). The number of dense segments changes from 20 to 40 in anaphase chromosomes of embryonic presomatic cells of *Cyclops kolensis* Lilljeborg 1901 (Grishanin 1995). These observations suggest that the condensation pattern of prediminution chromosomes in some way contributes to its specification for excision and elimination. The heterochromatin localization is strongly species-specific. Heterochromatin is localized in the telomeric area of *Cyclops divulsus* and in or near the centromeres and telomeres of *Cyclops furcifer* and *Mesocyclops edax*, but is evenly distributed throughout the chomosomes in *Cyclops strenuus strenuus* (Germany population)and *Cyclops kolensis* (Beermann 1977, Wyngaard and Chinnappa 1982, Grishanin 1995, Grishanin et al. 1996). Standiford (1989) compared C-banding patterns of *Acanthocyclops vernalis* Fischer, 1853 chromosomes before and after chromatin diminution to identify the heterochromatin regions eliminated during CD, and found that as a result of CD part of the heterochromatin of *Acanthocyclops vernalis* chromosomes cut out. Embryonic cells of German populations of *Cyclops strenuus strenuus* until fourth cleavage division showed clear separation of paternal and maternal chromosomes (Beermann 1977). Heterozygous females have two type of pronuclei: one with a set of heterochromatin-rich chromosomes and the other with a low heterochromatin set. CD totally eliminates a significant difference in the size between homologous chromosomes. In other words, the length of euchromatin part of chromosome is constant , while the length of the heterochromatic regions varies. Lecher et al. (1995) proposed that the heterochromatin segments that are excised in CD consist not only from highly repetitive DNA and might be considered as facultative heterochromatin. Subsequent studies have shownthat the eliminated DNA of *Cyclops kolensis* is composed ofmany direct and inverted repeats with a complex internal structure present within the same fragment (Degtyarev et al. 2004). The repetitive sequences (motifs) of *Cyclops kolensis* eliminated DNA have a mosaic structure consisting of submotifs (short repeats) and are distributed throughout the eliminated genome (Degtyarev et al. 2004). The study of inter simple sequence repeats (ISSR) of *Cyclops kolensis* showed that most of them are stored after CD (Zagoskin et al. 2008) Some sequences of eliminated DNA are selectively reduced during CD (Grishanin et al. 2006a, Zagoskin et al. 2008). The investigation of *Cyclops kolensis* rDNA before and after CD demonstrated a reduction of three hundred times of rRNA genes in the somatic cells line (Zagoskin et al. 2010). Comparative analysis of eliminated DNA of Moscow and Baikal populations of *Cyclops kolensis* showed a high level homology of repeats (97–98%) (Grishanin et al. 2006a). This means that, despite the huge number of generations that have passed since the divergence of Moscow and Baikal *Cyclops kolensis* population (at least 25 million), the sequence data has been under strong selection to not change.

Of special interest is the research on genome endoreduplication in cyclops with CD (Rash et al. 2008, Wyngaard et al. 2011). The mechanism of endoreduplication makes it possible to reverse the process of CD.

**Table 1.** Cytogenetic characteristics of Cyclopoida species with chromatin diminution (CD). **1** Akif’ev 1974 **2** Beermann 1959 **3** Beermann 1977 **4** Chinnappa 1980 **5** Dorward and Wyngaard 1997 **6** Einsle 1964 **7** Einsle 1975 **8** Einsle 1993 **9** Einsle 1994 **10** Einsle 1996 **11** Grishanin et al. 1996 **12** Grishanin et al. 2004 **13** Ivankina et al. 2013 **14** Kochina and Monchenko 1986 **15** Rasch and Wyngaard 2006 **16 **Rasch et al. 2008 **17** Semeshin et al. 2011 **18** Standiford 1989 **19** Wyngaard et al. 2011 **20** Zagoskin et al. 2010, nd= no data, PD/SC = DNA ratio of prediminuted germ cell nuclei and somatic cell nuclei.

Species	PD/ SC	n	CD time (cleavage division)	References
*Acanthocyclops incolotaenia* Mazepova, 1950	nd	nd	nd	13
*Acanthocyclops robustus* Sars G.O., 1863	nd	4	6	5,18
*Acanthocyclops vernalis* Fischer, 1853	nd	nd	5	1
*Apocyclops paramensis* Marsh, 1913	nd	nd	7	5
*Cyclops abyssorum* Sars GO, 1863	nd	nd	5	8
*Cyclops bohater* Kozminski, 1933	nd	nd	5	8
*Cyclops insignis* Claus, 1857	nd	nd	5	8
*Cyclops furcifer* Claus, 1857	2	11	6,7	3
*Cyclops heberti* Einsle, 1996	nd	nd	5	10
*Cyclops kikuchi* Smirnov, 1932	nd	11	nd	9,14
*Cyclops kolensis* Lilljeborg 1901	15.6–16.4	11	4	11,20
//------//-------//	11.2–12.4	11	4	17
//------//-------//	31–40	11	4	19
*Cyclops singularis* Einsle, 1996	nd	nd	4	10
*Cyclops strenuus divulsus* Lindberg, 1957	1.7	11	5	3
*Cyclops strenuus strenuus* Fisher, 1851	2.4	11	4	3
//------//------//	4	12	5,6	11
//------//------//	5.7	nd	nd	15
*Cyclops vicinus* Ulyanin, 1875	nd	11	nd	7,9
*Diacyclops galbinus* Mazepova 1950	11.9–13.2	nd	nd	13
*Diacyclops navus* Herrick, 1882	nd	nd	5	5
*Mesocyclops edax* Forbes, 1891	5.2–10.5	7	4	4,15,16
*Mesocyclops longisetus* Thiébaud, 1912	9.5	nd	6	5,15
*Mesocyclops longisetus curvatus* Dussart, 1987	14.6	nd	nd	15
*Metacyclops mendocinus* Wierzeiski, 1892	10	nd	nd	15
*Microcyclops varicans* Sars G.O., 1863	nd	nd	nd	2
*Paracyclops affinis* Sars G.O., 1863	1.75	nd	nd	12

## The mechanism of chromatin diminution in freshwater copepods

Beermann (1977) proposed that CD in cyclops involved the synthesis or activation of enzymes that initiate CD in presomatic cells during the prediminution interphase. In her opinion, the absence, inactivation or repression of diminution enzymes in germ line cells is sufficient to explain their failure to undergo CD. Beermann (1977) assumed that ectosomes in germ line cells contain either a non-specific repressor with such functions or a factor that induces the formation of such repressor. According to Beermann (1977), the mechanism for eliminating chromatin from the chromosomes of somatic cells is analogous to the mechanism of excising bacteriophage DNA from *Escherichia coli* Esherich, 1885 chromosomes. It involves looping of the eliminated region of chromosomes, recombination of homologous sites in the loop basement and joining of the chromosome fragments. This hypothesis is supported by the fact that the diploid chromosomes number before and after CD remains unchanged in the studied species of *Cyclops* Muller, 1776. It is also supported by the presence in embryonic cells of *Cyclops divulsus* and *Cyclops furcifer* of numerous chromatin rings 25–30 nm in diameter and 0.6–100 microns in length immediately after the beginning of diminution events irrespective of the localization of the eliminated chromosome regions (Beermann and Meyer 1980, Beermann 1984). Beermann explains the evolutionary changes affecting the localization and size of eliminated regions of chromosomes as a result of chromosome rearrangements, primarily deletions and duplications. The model of organization of the higher order chromatin loop (Mirkovitch et al. 1984) explains the data of Beermann and Meyer (Beermann and Meyer 1980, Beermann 1984). The domain-loops excised from cyclops chromosomes could be cut out in the base of loops at the site of Matrix Associated Regions (MAR). With this theory in mind it is now possible to complete the explanation of CD mechanism for *Cyclops kolensis* which was presented earlier (Akif’ev et al. 1998, 2002) and propose the following CD stages that occurs in the presomatic cells of cyclops:

1) Preparation for the reduction of a major part of the genome, which involves lengthening of the prediminution interphase and the appearance of G-bands in chromosomes prior to diminution. The G-bands might be involved in reprogramming the functionally active part of the genome through the mechanisms of DNA methylation and histone modifications of somatic cells chromosomes before CD;

2) Activation of the sites of chromosomes breaks during interphase of the cell cycle when CD occurs. It is probable that the chromosomes breakage sites are localized in regions associated with the nuclear matrix;

3) Cutting at chromosomes breakage sites. Immediately after cutting, the chromosome DNA strand is restored;

4) Compacting of the excised DNA and formation of a pore-free membrane around it to produce granules;

5) Degradation of the granules of excised DNA during 2–3 subsequent divisions.

Thus, the CD process, presumably, involve many genes.

## Biological role and evolutionary significance of CD

CD is unique in producing a dramatic reorganization of the entire nuclear genome during a relatively short period of ontogeny. During CD large regions of heterochromatin are removed from chromosomes of the somatic cells line. Prior to CD, presomatic cells of cyclops in interphase have a nuclear structure that is highly ordered in terms of the spatial distribution of eu- and heterochromatin. There is a strong opinion based on numerous facts that silent genes are localized in the heterochromatin compartments at the nuclear periphery, whereas active genes are located in the central part (Dillon 2004, Meaburn and Misteli 2007, Schofer and Weipoltshammer 2008). Hollick et al. (1997) showed that rapid genome reorganization is associated with repetitive DNA, its methylation and insertions of a transposable element. Therefore, the excision of heterochromatin from chromosomes during CD can remove genes, change their position, and through the mechanisms of DNA methylation and histone modifications modify their regulatory status. Moreover, presumably, the excision of heterochromatin segments by CD will decrease the distance between many previously distantly located genes. This is expected to increase the amount ofinterference from crossover exchanges and decrease the number of possible recombinations, which in turn is expected to reduce adaptiveness.There is an alternative, albeit radical evolutionary solution of reducing the number of recombinations during meiosis – an absence of chiasmata. It’s usual for Cyclopoidae species to have achiasmatic meiosis (Chinnappa 1980, Grishanin et al. 2005). However some cryptic species have meiosis with well-defined chiasmata, variable genome and maybe capable of rapid evolutionary changes (Grishanin et al. 2005, 2006b).

Monchenko (2003) emphasizes that macromorphological traits are of little importance in the speciation of cyclops, which has many cryptic species. Cryptic speciation is apparently common in the Cyclopoida. The study *Acanthocyclops vernalis* revealed a complex population structure of this species, where some populations not only have different cytogenetic characteristics, but also show a partial or complete reproductive isolation from other populations (Grishanin et al. 2005, 2006c). These data suggest that these populations can be considered as cryptic species (Dodson et al. 2003). Comparative study of the Cyclopoida species (*Acanthocyclops vernalis*, *Cyclops insignis*, *Cyclops kolensis*, *Cyclops strenuus strenuus*) has revealed that a large-scale rearrangement of the genome has arisen in this suborder without any visible morphological changes. Evolutionary events that have involved changes in genome size but not changes in chromosome number are evidenced by multiple genome size differences within the genera *Mesocyclops* Sars, 1913 and between populations of *Thermocyclops crassus* Fischer, 1853 (Table 2) (it’s probably by mechanisms of endoreduplication); the chromosome polymorphism observed in *Cyclops strenuous strenuus* like gonomery (Beermann 1977), the cytogenetics differences observed between Russian and Germany populations of *Cyclops strenuous strenuus*; and the presence and absence of CD for Russian and German populations of *Cyclops insignis* (Grishanin and Akifiev 2000).The molecular data complete the cytogenetics pattern. The high level of conservation of the *Cyclops kolensis* genome, the complex structure of its eliminated DNA, and the selective removal of some sequences, (Degtyarev et al. 2004, Grishanin et al. 2006a, Zagoskin et al. 2010) suggests a special role of eliminated DNA in its development and evolution. So, CD process should be considered as an evolutionary innovation that leads directly to the appearance of cryptic Cyclopoida species that are distinguished by the occurrence or lack of CD, by peculiarities of the CD process and by other cytogenetic characteristics. The mechanism of cryptic speciation is not known yet, but it is likely that hybrids of cyclops with CD and without CD will fail as a result of a compromised ability to regulate CD. Disruption of the coordinated network of genes that control each of the above-proposed stages of CD will inevitably lead to the disturbance of the CD process and, as a result, to errors in the processes of development and differentiation of the organism that will most likely cause the death for the given organism. Consequently, the occurrence of CD in *Cyclops* evolution should automatically lead to the emergence of a new species. To confirm this hypothesis, it would be of interest to cross the German population of *Cyclops insignis*, whichhave CD and the Russian population of *Cyclops insignis*, in which CD absent.

**Table 2.** Cytogenetic characteristics of *Thermocyclops crassus* Fischer, 1853 and some species of *Mesocyclops* Sars, 1913. **1** Wyngaard and Rasch 2000 **2** Rasch and Wyngaard 2006 **3** Grishanin et al. 2004 **4** Grishanin 2008, SC = somatic cell nuclei.

Species	1C(pg) SC	n	CD	References
*Mesocyclops edax* Forbes, 1891	1.47	7	present	1,2
*Mesocyclops ruttneri* Kiefer, 1981	0.72	7	no CD	1
*Mesocyclops leuckarti* Claus, 1857	0.38	7	no CD	1
*Mesocyclops woutersi* van de Velde, 1987	0.38	no data	no CD	1
*Thermocyclops crassus* (russian population)	1.2	7	no CD	3,4
*Thermocyclops crassus* (vietnamese population)	0.42	no data	no CD	1

The detection of polyploid cells in some *Cyclops* species raises another side of the biological significance of CD (Grishanin et al. 1996). With the conventional notion of polypoidy arising during or before conception, the functional advantage of having multiple copies of some

DNA sequences is offset by the necessity of replicating and maintaining multiple copies of much additional genetic material that will never be required in differentiated somatic cells. An alternative but more economical path to the same end is to eliminate unused DNA from the somatic cells line genome during CD and then to repeatedly amplify the genome of somatic cells.

Based on the above reasoning we can make the following assumptions about biological role of CD:

1. Chromatin diminution is an alternative form of regulation of cell differentiation during which there is a total loss of mostly redundant DNA, while in the vast majority of eukaryotes part of the genome is inactivated through heterochromatinization;

2. CD coevolves with polyploidy to regulate gene dosage in somatic tissues;

3. CD is a tool for adaptation to changing environmental conditions;

4. CD is a mechanism for regulating the rate of evolutionary processes.

Thus, even a brief acquaintance with the facts established recently permits a conclusion that the phenomenon of CD is a unique tool to study the eukaryotic nucleus organization and some questions of evolution.
